# Development of a Whole Organism Platform for Phenotype-Based Analysis of IGF1R-PI3K-Akt-Tor Action

**DOI:** 10.1038/s41598-017-01687-3

**Published:** 2017-05-17

**Authors:** Chengdong Liu, Wei Dai, Yan Bai, Changfeng Chi, Yi Xin, Gen He, Kangsen Mai, Cunming Duan

**Affiliations:** 1 The Key Laboratory of Mariculture, Education Ministry of China and College of Fisheries, Ocean University of China, Qingdao, 266003 China; 2 Department of Molecular, Cellular and Developmental Biology, University of Michigan, Ann Arbor, MI 48109 USA; 3 National Engineering Research Center for Marine Facilities Aquaculture, School of Marine Science and Technology, Zhejiang Ocean University, Zhoushan, 316022 China; 4 Department of Molecular, Cellular and Developmental Biology, University of California, Santa Barbara, CA 93106 USA

## Abstract

Aberrant regulation of the insulin-like growth factor (IGF)/insulin (IIS)-PI3K-AKT-TOR signaling pathway is linked to major human diseases, and key components of this pathway are targets for therapeutic intervention. Current assays are molecular target- or cell culture-based platforms. Due to the great *in vivo* complexities inherited in this pathway, there is an unmet need for whole organism based assays. Here we report the development of a zebrafish transgenic line, *Tg*(*igfbp5a*:*GFP*), which faithfully reports the mitotic action of IGF1R-PI3K-Akt-Tor signaling in epithelial cells in real-time. This platform is well suited for high-throughput assays and real-time cell cycle analysis. Using this platform, the dynamics of epithelial cell proliferation in response to low [Ca^2+^] stress and the distinct roles of Torc1 and Torc2 were elucidated. The availability of *Tg*(*igfbp5a*:*GFP*) line provides a whole organism platform for phenotype-based discovery of novel players and inhibitors in the IIS-PI3K-Akt-Tor signaling pathway.

## Introduction

The evolutionarily ancient insulin-like growth factor (IGF)/insulin (IIS) hormonal pathway plays key roles in regulating development, growth, metabolism, and aging. The IIS system is also implicated in stem cell biology, neurodegenerative diseases, diabetes, and cancers^1–4^. In vertebrates, the ancestral IIS pathway has diverged into the insulin and IGF signaling pathways^5^. The biological actions of IGFs and insulin are mainly mediated through the IGF1 receptor (IGF1R) and insulin receptor (InsR), respectively. A simplified view is that the insulin-InsR pathway is critical in regulating metabolism, while the IGFs-IGF1R pathway plays a more important role in regulating growth and longevity^5^. In many cell types, IGFs and insulin are strong activators of the phosphatidylinositol-4,5-bisphosphate 3-kinase (PI3K)-Akt pathway^6–8^. Ligand binding activates the respective receptor, which in turn phosphorylates an insulin receptor substrate (IRS). The tyrosine phosphorylated IRS binds to and activates PI3K^9^. PI3K increases PIP3, while PTEN opposes this reaction. Elevated PIP3 recruits Akt to the plasma membrane where it is phosphorylated at T308 by PDK1. Target of Rapamycin (TOR), a serine/threonine kinase, exists in the rapamycin-sensitive TORC1 complex and the rapamycin-insensitive TORC2 complex^10^. While TORC2 phosphorylates Akt at S473 and increases Akt activity^11^, TORC1 activity is under the regulation of Akt. Akt-mediated phosphorylation of TSC2 inhibits its GAP activity, allowing GTP-bound RHEB to stimulate TORC1 activity^12^. Additionally, Akt phosphorylates PRAS40, which relieves its inhibition of TORC1 catalytic activity^13^.

The IIS-PI3K-AKT-TOR signaling pathway is dysregulated in many human diseases, including growth disorders, diabetes, and obesity^14^. Hyper-activation of PI3K, AKT, and/or mTOR contributes to the initiation and progression of tumors^14, 15^. In fact, the IIS-PI3K-AKT is one of the most frequently mutated signaling pathways in several types of cancer^4^. For instance, multiple mutations have been identified in genes belonging to this pathway in colon cancers, including mutations that increase *IGF2* gene expression, increase PIK3CA and PIK3R1 activities, and reduce *PTEN* expression^16^. The *IRS2* gene is also frequently gained in colon cancer^17^ and has been proposed as a potential colorectal cancer “driver” oncogene^18^. In glioblastoma, the class IA PI3K p110α subunit gene is the most mutated gene^19^. Mutations in the *TSC* gene have been shown to lead to a tuberous sclerosis complex, which exhibits as benign lesions and increases the risk of renal cell carcinoma^14^. As such, major components of the IIS-PI3K-Akt pathway have targeted as points of therapeutic intervention. A number of assays have been developed, and potent inhibitors for IGF1R/InsR, PI3K, AKT, PTEN, and mTOR have been discovered^20, 21^. Most, if not all, available assays are molecular target- or cell culture-based platforms. We now understand that there are tremendous complexities in the IIS-PI3K-AKT-mTOR signaling pathway *in vivo*, ranging from crosstalk with other hormones/growth factors, the availability of local nutrients and oxygen, to various feedback mechanisms. IGFs in particular are bound to a family of high-affinity IGF binding proteins (IGFBPs) in the extracellular environments. The IGF/IGFBP complexes prolong the half-lives of IGFs and buffer the potential hypoglycemic effects of high concentrations of IGFs in circulation. Locally expressed IGFBPs alter IGF availability and biological activity by modulating their interaction with the IGF1R^22^. These complexities cannot be captured by a molecular target- or cell culture-based assay. The existence of various redundant and compensatory mechanisms in an organism cannot be assayed *in vitro*, either.

Zebrafish is an ideal model organism for whole organism-based chemical biology screens^23^. Zebrafish are vertebrates and share a high degree of genetic and anatomical homology with humans. Zebrafish spawn and produce large numbers of eggs daily. It is easy to subject the tiny, free living, and transparent embryos and larvae to various doses of chemicals and to observe and score morphological and physiological changes. The chemicals can be added conditionally during a specific developmental stage(s) and/or at different concentrations, thus adding a temporal dimension and making this a tunable system^23^. With the rapid advances in genetic tools such as CRISPR/Cas9 genome editing technology and Tol2 transposon-BAC transgenesis, it is feasible to genetically engineer mutant and transgenic zebrafish lines that can mimic certain human disease conditions^24^. Recent adoption of automated screening platforms has further increased the power of the zebrafish model in chemical biology/chemical genomics screens^23^.

In a recent study, we have shown that the IIS-PI3K-Akt signaling pathway is activated and is required in the proliferation of a group of epithelial cells, known as Na^+^-K^+^-ATPase-rich (NaR) cells, under low [Ca^2+^] stress^25^. Zebrafish live in aquatic environments and must import Ca^2+^ from the surrounding water using NaR cells to maintain their calcium homeostasis^26^. These cells are functionally equivalent to the Ca^2+^-transporting epithelial cells in human intestine and kidney and contain all the molecular players for transcellular Ca^2+^ transport, including Trpv5/6, an epithelial Ca^2+^ channel^27^. In the adult stage NaR cells are located in the gills (a major osmoregulatory organ in adult fish) and intestine. In the embryonic and larval stages, however, they are located on the surface of the yolk sac skin^26^, making them easily accessible for drug treatment and observation. When exposed to a low [Ca^2+^] environment, NaR cells undergo robust and unrestrained proliferation. This is entirely due to the activation of IGF1R-PI3K-Akt signaling in these cells^25^. This has provided an excellent inducible system to investigate the regulatory mechanisms of IGF1R-PI3K-Akt-Tor pathway in an *in vivo* whole animal setting. However, visualization of NaR cells by *in situ* hybridization and measuring their number manually is not only labor intensive, but also prevent real time analysis of the NaR cell proliferative response.

In this study, we have developed a stable zebrafish transgenic line by labeling NaR cell with GFP. These transgenic larvae faithfully report the action of IGF1R-PI3K-Akt signaling and are well suited for high-throughput and real-time cell cycle analysis. Using this platform, the dynamics of NaR cell proliferation in response to low [Ca^2+^] stress and the distinct roles of Torc1 and Torc2 in this process were elucidated.

## Results

### Low [Ca^2+^] stress induces NaR cell proliferation and a concordant increase in *igfbp5a* mRNA levels

**Figure 1:**
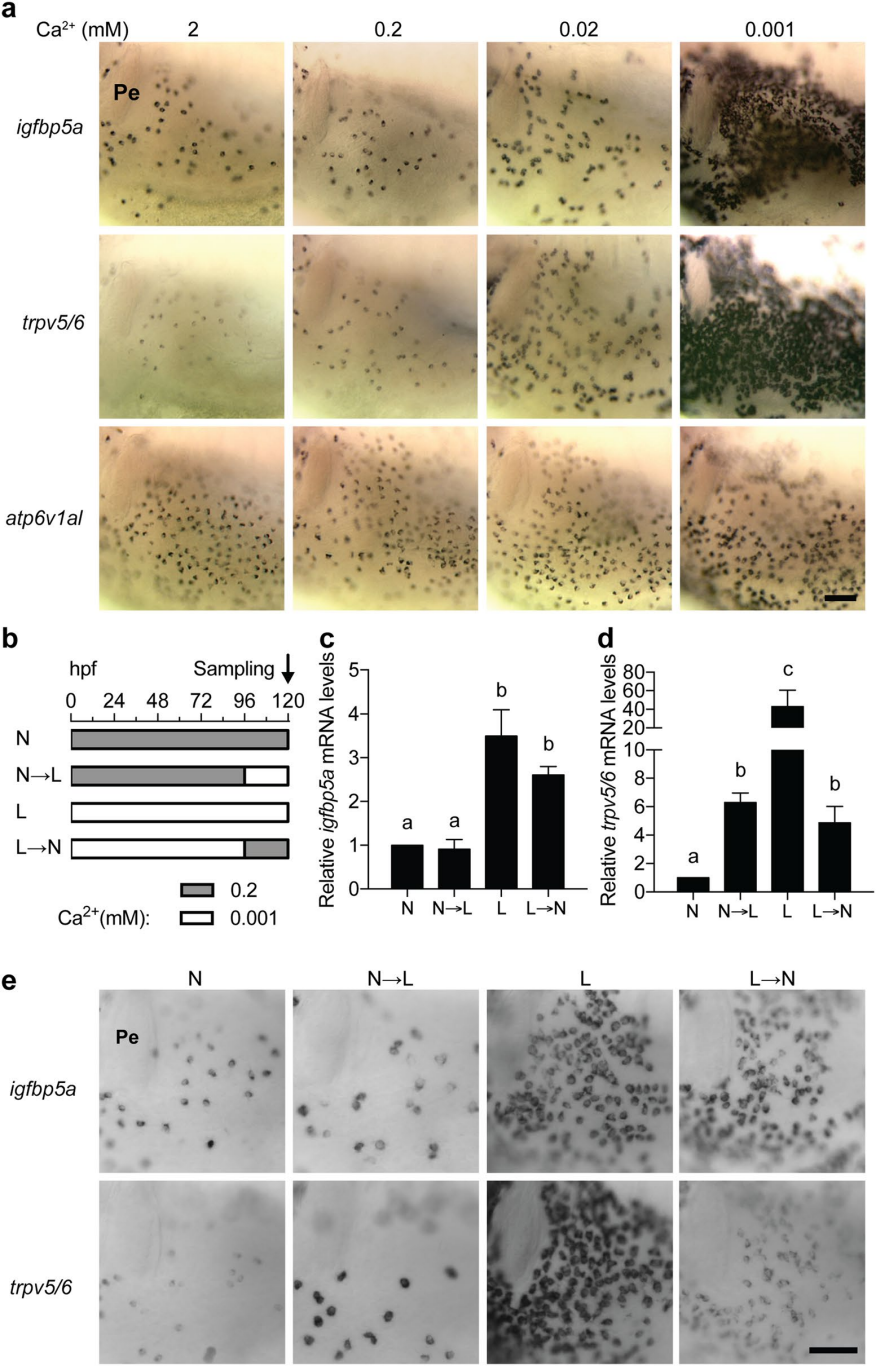
The *igfbp5a* gene is specifically expressed in NaR cells. (**a**) Wild type zebrafish embryos were raised in embryo rearing solution containing the indicated [Ca^2+^] from 0 to 120 hpf (hours post fertilization) and analyzed by whole-mount *in situ* hybridization using the indicated probes. Images shown are the yolk sac region. Scale bar = 50 μm. Unless specified otherwise, all *in situ* hybridization images shown hereafter are lateral views, anterior to the left and dorsal up. (**b**–**e**) The *trpv5*/*6* and *igfbp5a* genes respond differentially to [Ca^2+^] changes. The experimental design is shown in (**b**). The mRNA levels of *igfbp5a* (**c**) and *trpv5*/*6* (**d**) were measured by qPCR and normalized by the *β-actin* mRNA levels. Data shown are mean ± SEM, n = 3. Different letters indicate significant differences at *p* < 0.05. (**e**) The larvae described in (**b**) were analyzed by *in situ* hybridization using the indicated probes. Representative images are shown.

In a previous study, we have reported that *igfbp5a* mRNA is specifically expressed in NaR cells and that whole body *igfbp5a* mRNA levels are a good indicator of NaR cell number in larval zebrafish^25^. The *trpv5*/*6* mRNA is also specifically expressed in NaR cells. In fact, it is considered as a NaR cell marker gene^25^. We therefore wondered which gene is a better indicator of NaR cell number. Moreover, the time-course effects of low [Ca^2+^] on *igfbp5a* and *trpv5*/*6* expression were not examined and it is unclear whether the low [Ca^2+^] effects are reversible. To answer these questions, wild type zebrafish embryos were raised in embryo rearing solutions containing various concentrations of [Ca^2+^] from 0 to 120 hpf. Compared to the larvae raised in normal [Ca^2+^] (0.2 mM) and high [Ca^2+^] (2 mM) solution, those raised in 0.02 and 0.001 mM [Ca^2+^] solutions had many more *igfbp5a* mRNA- and *trpv5*/*6* mRNA-expressing NaR cells (Fig. 1a). The increase was most robust in the 0.001 mM [Ca^2+^] group (Fig. 1a). Changes in [Ca^2+^] caused limited changes in the number of HR (H^+^-ATPase-rich) cells, which was labeled by *atp6v1al* mRNA expression (Fig. 1a). When analyzed by qPCR, the *igfbp5a* mRNA levels in the L group (i.e., 0.001 mM [Ca^2+^]) were 3.5-fold greater than the N group (i.e., 0.2 mM [Ca^2+^]) (Fig. 1b and c). The levels of *trpv5*/*6* mRNA in the L group was 43-fold greater than those of the N group (Fig. 1d). Switching from the normal [Ca^2+^] to the low [Ca^2+^] solution (i.e., N → L group) resulted in a 6.3-fold increase in the *trpv5*/*6* mRNA levels (Fig. 1d), while it did not change the *igfbp5a* mRNA levels (Fig. 1c) or the NaR cell density (Fig. 1e). Conversely, switching from the low [Ca^2+^] to normal [Ca^2+^] (i.e., L → N group) significantly reduced the *trpv5*/*6* mRNA levels (Fig. 1d) but had no effect on *igfbp5a* mRNA levels (Fig. 1c) or NaR cell density (Fig. 1e). Therefore, while low [Ca^2+^] stress increases *igfbp5a* and *trpv5*/*6* mRNA levels, the two genes are differentially regulated.

**Figure 2:**
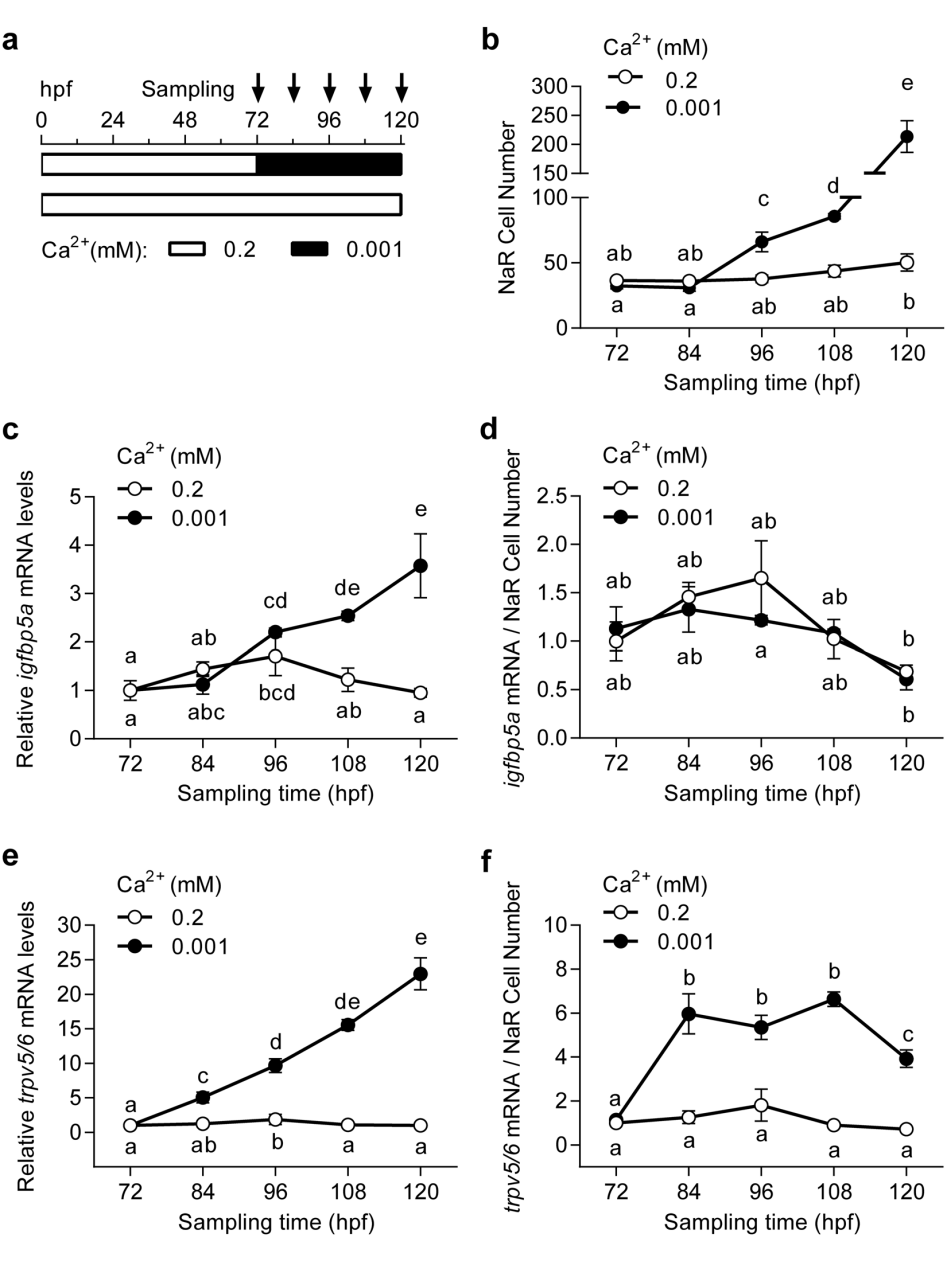
The *igfbp5a* mRNA levels are correlated with NaR cell number. (**a**) The experimental design. (**b**) Larvae described in (**a**) were analyzed by *in situ* hybridization for *igfbp5a* mRNA expression. The NaR cell number on one side of yolk sac was manually counted. (**c**) Total *igfbp5a* mRNA levels measured by qRT-PCR and by normalized by *β-actin* mRNA levels. (**d**) The *igfbp5a* mRNA levels shown in (**c**) are normalized by NaR cell number shown in (**b**). (**e**) The *trpv5*/*6* mRNA levels measured by qRT-PCR and by normalized by *β-actin* mRNA levels. (**f**) The *trpv5*/*6* mRNA levels shown in (**e**) are normalized by NaR cell number shown in (**b**). All data shown above are mean ± SEM, n = 3. Different letters indicate significant differences at *p* < 0.05.

Next, we examined the effect of low [Ca^2+^] stress in different developmental stages. Low [Ca^2+^] treatment during the embryonic and early larval stage (i.e., from 0 to 48 and from 0 to 72 hpf) significantly increased the *trpv5*/*6* mRNA levels (Supplemental Fig. S1a), while did not change the *igfbp5a* mRNA levels and NaR cell number in these stages (Supplemental Fig. S1b,c). The basal levels of *trpv5*/*6* and *igfbp5a* mRNA increased from 48 to 72 hpf regardless of water [Ca^2+^], reflecting a developmental increase^25^. This result suggests that low [Ca^2+^] stress stimulates *trpv5*/*6* expression in both embryonic and larval stages, while it increases *igfbp5a* expression only in the larval stage. We next mapped the time window of the *igfbp5a* responsiveness by subjecting the embryos/larvae to low [Ca^2+^] stress at various time points (Supplemental Fig. S2a). Low [Ca^2+^] treatment of all time points significantly increased *trpv5*/*6* mRNA expression and the magnitude of increases is proportional to the lengths of treatment time (Supplemental Fig. S2b). However, significant increases in *igfbp5a* mRNA levels were seen only in those treated 84 hpf or earlier (Supplemental Fig. S2c). In fact, low [Ca^2+^] treatment from 72 to 120 hpf resulted in the same degree of increases in *igfbp5a* mRNA levels as those treated from 0 to 120 hpf (Supplemental Fig. S2c). Next, larvae were transferred from normal to low [Ca^2+^] solution at 72 hpf and sampled every 12 hours thereafter (Fig. 2a). In the normal [Ca^2+^] group, the NaR cell number showed modest and gradual increase (Fig. 2b). In the low [Ca^2+^] group, a significant increase was detected at 96 hpf. At 120 hpf, the NaR number was 4.2-fold of the normal [Ca^2+^] group (Fig. 2b). The *igfbp5a* mRNA levels in the low [Ca^2+^] were 3.8-fold over the normal [Ca^2+^] group (Fig. 2c). When normalized by NaR cell number, however, the *igfbp5a* mRNA levels were similar between the two groups (Fig. 2d), indicating that the increase in *igfbp5a* mRNA levels is mainly due to the increased NaR cell number. The changes in *trpv5*/*6* mRNA levels were more profound and rapid. A 4.1-fold significant increase was defected after 12 hours low [Ca^2+^] treatment (Fig. 2e). At 120 hpf, the *trpv5*/*6* mRNA levels increased to 23-fold of the normal [Ca^2+^] group (Fig. 2e). When normalized by NaR cell number, the *trpv5*/*6* mRNA levels in the low [Ca^2+^] group were still 4~6-fold higher than those of the normal [Ca^2+^] group (Fig. 2f), suggesting that the elevated *trpv5*/*6* mRNA levels are due to the increased NaR cell number as well as increased *trpv5*/*6* mRNA expression per cell.

### Generation and characterization of stable *Tg*(*igfbp5a*:*GFP*) zebrafish

**Figure 3:**
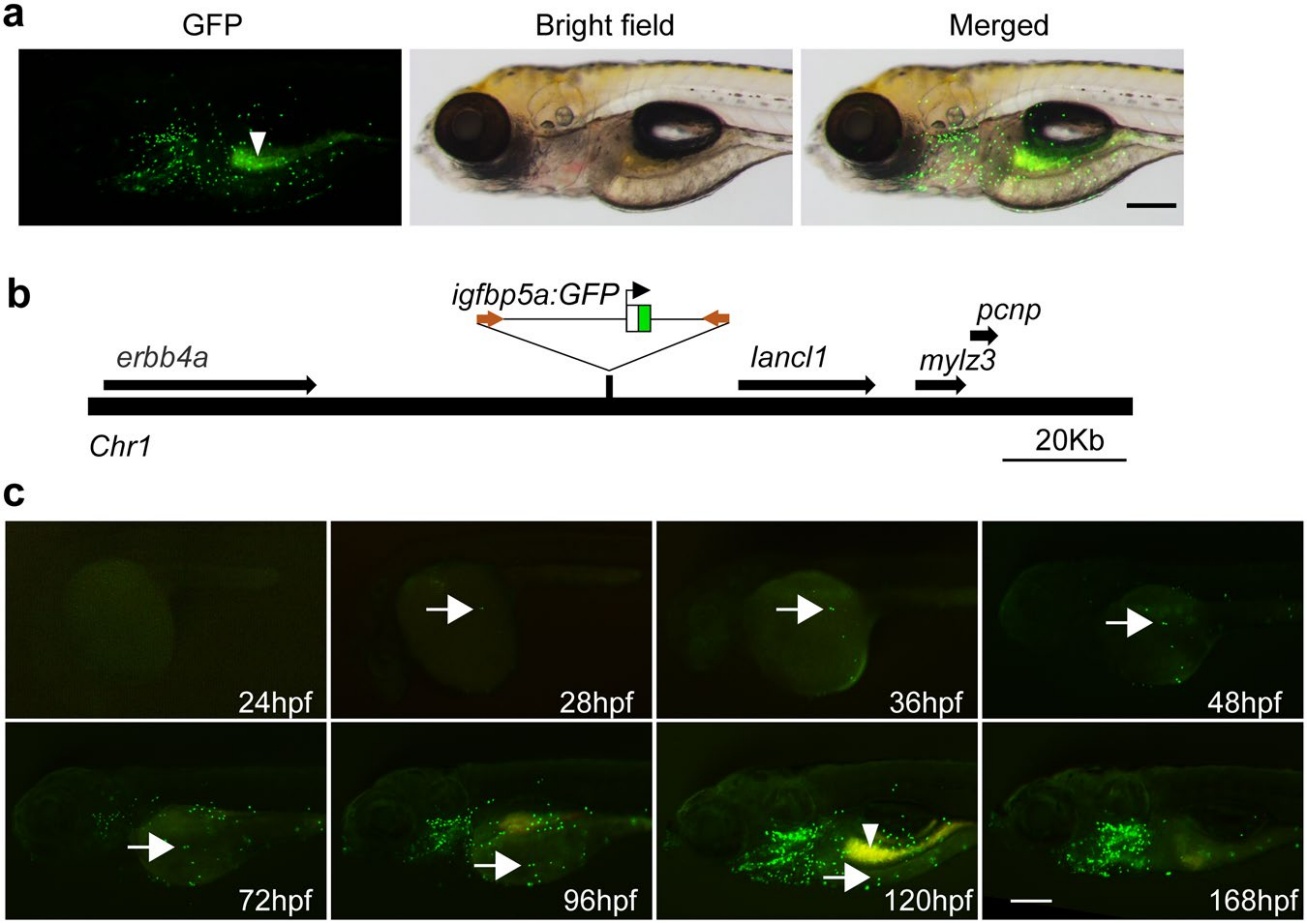
Characterization of the stable *Tg*(*igfbp5a*:*GFP*) fish line. (**a**) Lateral view of a 120 hpf transgenic *Tg*(*igfbp5a*:*GFP*) larva. The GFP-labeled cells are located in the yolk sac region and gills. Auto-fluorescence is indicated by an arrowhead. Scale bar = 0.2 mm. (**b**) Schematic diagram showing the genomic integration site. (**c**) Developmental pattern of GFP-labeled cells. Arrows indicate the emergence of GFP-positive cells and its subsequent location in the indicated stages. Auto-fluorescence is indicated by an arrowhead.

Because the *igfbp5a* mRNA levels are better correlated with NaR cell number changes, we chose to use its promoter to generate a GFP reporter fish line. Initial attempts using plasmids covering various *igfbp5a* 5′ UTR regions failed to mimic the endogenous *igfbp5a* expression pattern. We next used a BAC clone (DKEYP-56B7, EMBL accession: BX321894.8), which contains 113 kb upstream and 60 kb downstream of *igfbp5a* (Supplemental Fig. S3a). iTol2 cassette was introduced, and a GFP cassette with stop codon was used to replace the *igfbp5a* sequence starting from the start codon to the end of the first exon (Supplemental Fig. S3a). The validated *BAC*(*igfbp5a*:*GFP*) (Supplemental Fig. S3b,c) was injected into cells of 1-cell stage embryos. F0 larvae showed mosaic GFP expression in the yolk sac and gills (Supplemental Fig. S3d). F0 individuals were raised, crossed, and a stable fish line was obtained. In the stable *Tg*(*igfbp5a*:*GFP*) larvae, GFP-expressing cells were found on the yolk sac skin and gills in a salt-and-pepper pattern (Fig. 3a). The integration site was mapped to a region between *erbb4a* and *lancl1*, where there is no known gene (Fig. 3b). The GFP-labeled cells were first detected at 28 hpf in the yolk sac region (Fig. 3c). The number of GFP-labeled cells increased as the embryos grew. At 72 hpf, GFP-labeled cells also began to appear in the gill filament regions. At 168 hpf, most GFP-labeled cells disappeared from the yolk sac and became distributed in the gills (Fig. 3c). This pattern resembles the reported NaR cell developmental pattern^25, 28^.

**Figure 4:**
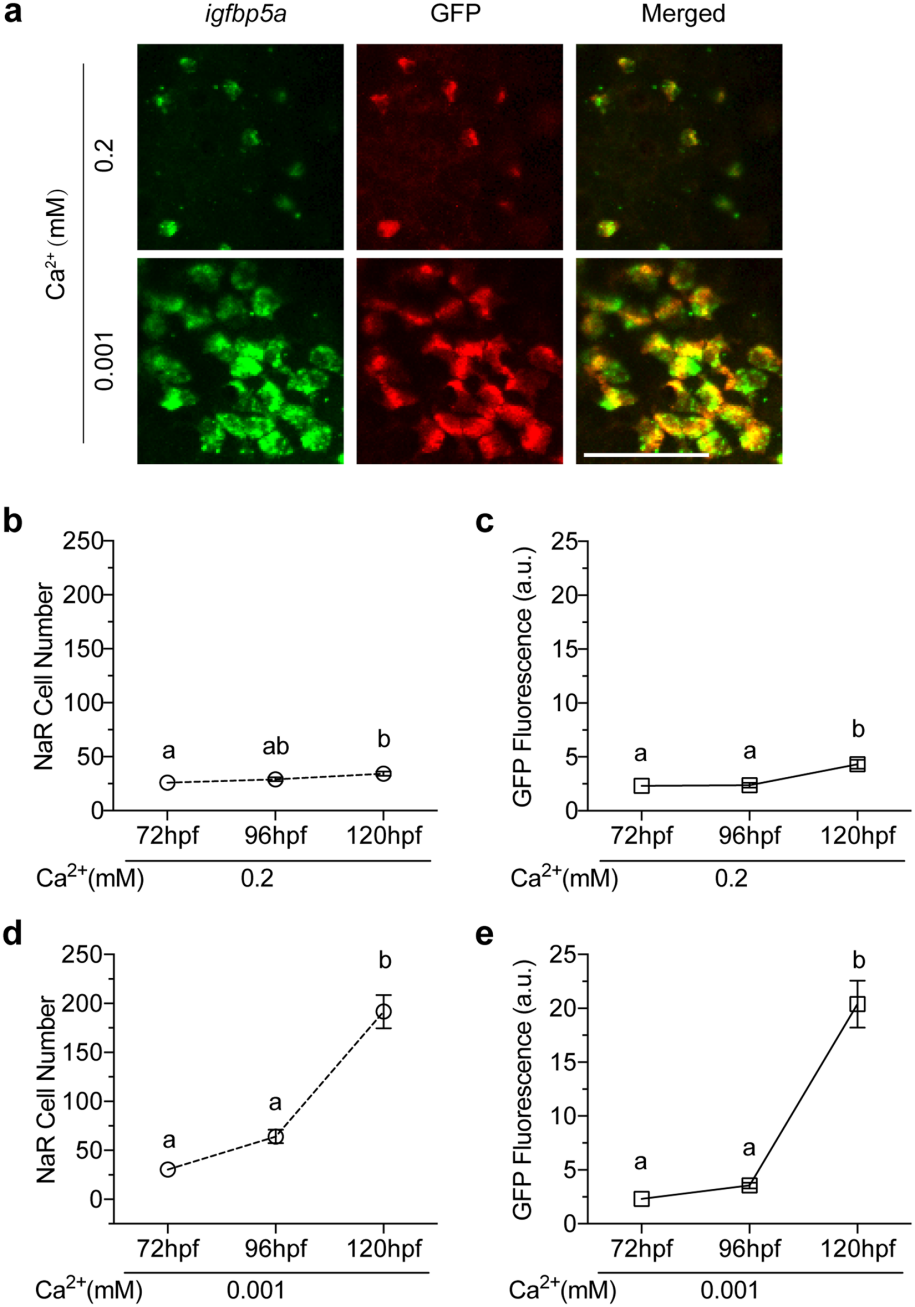
*Tg*(*igfbp5a*:*GFP*) fish faithfully recapitulates low [Ca^2+^] stress-induced NaR cell proliferation. (**a**) The GFP transgene is specifically expressed in NaR cells. *Tg*(*igfbp5a*:*GFP*) larvae (72 hpf) were transferred to embryo rearing solution containing the indicated [Ca^2+^]. 48 hours later, they were analyzed by *in situ* hybridization for *igfbp5a* mRNA expression (green, left panels) and GFP immunostaining (red, middle panel). Merged views are shown in the right panels. Scale bar = 50 μm. (**b**,**c**) Changes in NaR cell number **(b)** and GFP fluorescence intensity **(c)** under normal [Ca^2+^] at the indicated time points. Values shown are mean ± SEM. n = 7. Different letters indicate significant differences at *p* < 0.05. (**d**,**e)** Changes in NaR cell number **(d)** and GFP fluorescence intensity **(e)** under low [Ca^2+^] at the indicated time points. Values shown are mean ± SEM. n = 11. Different letters indicate significant differences at *p* < 0.05.

Double-label *in situ* hybridization and GFP immunostaining analysis revealed that the GFP-positive cells are indeed NaR cells because they expressed both *igfbp5a* (Fig. 4a) and *trpv5*/*6* mRNA (Supplemental Fig. S4a). Next, FACS cell sorting experiments were carried out and the levels *igfbp5a* and *trpv5*/*6* mRNA were measured by qPCR. Compared with GFP-negative cells, the *igfbp5a* and *trpv5*/*6* mRNA were highly enriched in the GFP-positive cells (Supplemental Fig. S4b,c). Moreover, low [Ca^2+^] treatment resulted in a further increase in the levels of *trpv5*/*6* mRNA (Supplemental Fig. S4c). These data suggest that the GFP-expressing cells are indeed NaR cells.

### *Tg*(*igfbp5a*:*GFP*) fish recapitulates low [Ca^2+^]-induced NaR cell proliferation

Next, *Tg*(*igfbp5a*:*GFP*) larvae were transferred to the low [Ca^2+^] solution from 72 hpf to 120 hpf. Low [Ca^2+^] treatment markedly increased the number and size of GFP-labeled cells (Fig. 4a). Compared to a modest increase in NaR cells in the normal [Ca^2+^] group (Fig. 4b), a robust and highly significant increase was observed in the low [Ca^2+^] group (Fig. 4d). At the end of the 48 h treatment, the NaR cell number increased to 6.4-fold over the beginning value (Fig. 4d). We also quantified GFP fluorescence intensity. The changes in GFP fluorescence intensity were essentially the same as the NaR cell number changes (Fig. 4c and e). Correlational analysis showed a highly significant correlation between the GFP fluorescence intensity data and NaR cell number (Supplemental Fig. S5). These data suggest that *Tg*(*igfbp5a*:*GFP*) faithfully reports low [Ca^2+^]-induced NaR cell proliferation, and the NaR cells can be determined by automated measurement of GFP fluorescence intensity.

**Figure 5:**
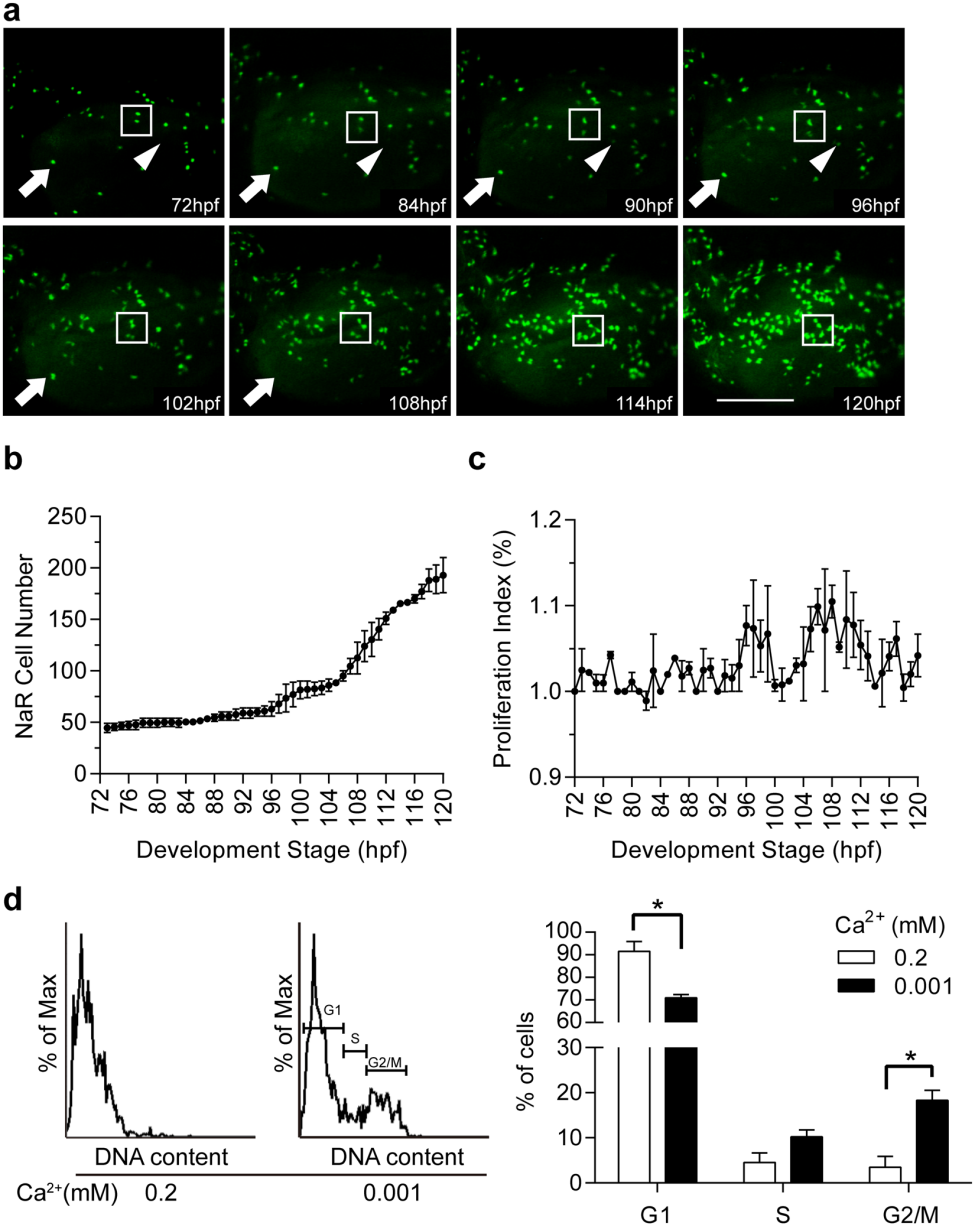
Dynamics of low [Ca^2+^]-induced NaR cell proliferation. *Tg*(*igfbp5a*:*GFP*) larvae (72 hpf) were transferred to the embryo rearing solution containing 0.001 mM [Ca^2+^]. Images were recorded from the same individual hourly and NaR cells numbers were determined. (**a**) Representative images at the indicated time points. The box shows two GFP-expressing NaR cells going through two divisions. The arrow indicates a NaR cell disappearing between 102–108 hpf. The arrowhead indicates the appearance of a new GFP-expressing cell around 86–90 hpf. (**b**) Average NaR cell number at the indicated time points. Values are mean ± SEM, n = 2. (**c**) NaR cell proliferation index at the indicated time periods. NaR cell proliferation index (%) = [(N_t_ − N_t−1_)/N_t−1_] * 100. N_t_ is the NaR cell number at the time of interest. N_t−1_ is the NaR cell number an hour earlier. (**d**) Cell cycle analysis results. Left panel is a representative cell cycle histogram. Quantitative results are shown in the right panel. Values shown are mean ± SEM, n = 3, *p < 0.05.

Time-lapse analysis showed that many GFP-labeled NaR cells underwent two rounds of cell divisions during the 48 hour treatment period (Supplemental Video 1). The first division was completed by 96 hpf (Fig. 5a). The second was completed by 114 hpf (Fig. 5a). Although occasionally one or two new GFP-labeled cells emerged and a few disappeared from the imaging field, a vast majority of newborn GFP cells were derived from pre-existing GFP-labeled cells (Fig. 5a). Consistent with the time-lapse data, the average NaR cell number showed a notable increase around 96 hpf and the increase sped up from 104 hpf until the end of the experiment (Fig. 5b). The proliferation index showed two major peaks. The first occurred around 96 hpf (i.e., 24 h treatment) and the second around 108 hpf (12 h later). After that, the proliferation index remained greater than 1, indicating continuous elevated proliferation rate (Fig. 5c). FACS analysis results showed that 18.3% NaR cells were in M/G2 phase in the low [Ca^2+^] group at 120 hpf, while only 3.4% NaR cells was in M/G2 phase in the normal [Ca^2+^] group (Fig. 5d). Likewise, the percentages of NaR cells in G1 phase were 71% and 91.6% in the low and normal [Ca^2+^] groups, respectively. These findings suggest that low [Ca^2+^] stress activates a mitotic program in pre-existing NaR cells.

### *Tg*(*igfbp5a*:*GFP*) fish report IGF1R-PI3K-Akt-Tor signaling-regulated cell proliferation

**Figure 6:**
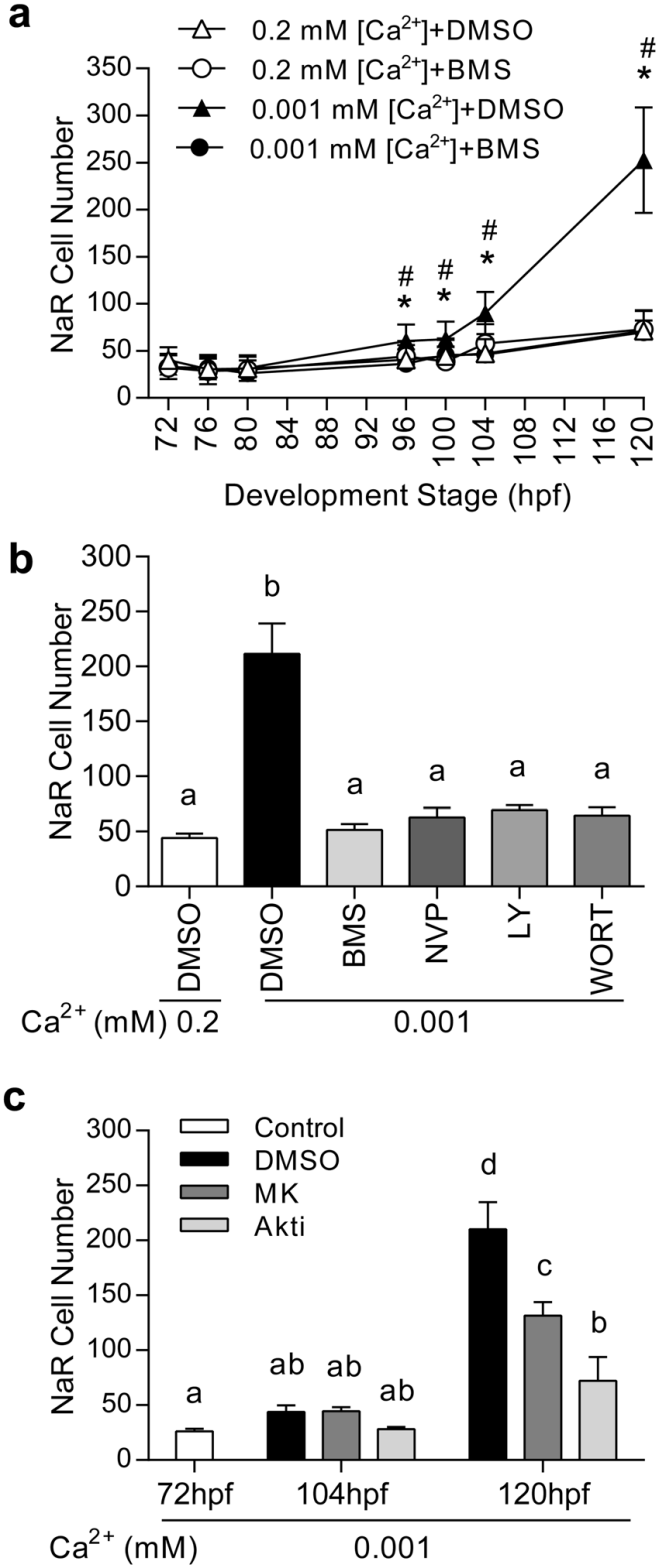
*Tg*(*igfbp5a*:*GFP*) fish faithfully recapitulates the mitotic action of IGF1R-PI3K-Akt-Tor signaling. (**a**) Low [Ca^2+^] stress induces NaR proliferation via IGF1R signaling. *Tg*(*igfbp5a*:*GFP*) larvae (72 hpf) were transferred to the indicated solutions. GFP fluorescence intensity was measured at the indicated time points. NaR cell number was calculated and shown. Value are mean ± SEM, n = 10–14. * indicates significant differences (*p* < 0.05) between the Low [Ca^2+^] + BMS group and the Low [Ca^2+^] + DMSO group at the same time point. # indicates significant differences (*p* < 0.05) between the Low [Ca^2+^] + DMSO group and the normal [Ca^2+^] + DMSO group at the same time point. (**b**) *Tg*(*igfbp5a*:*GFP*) larvae (72 hpf) were treated as described in (**a**) and NaR cell numbers were measured at 120 hpf. Inhibitors used are BMS-754807 (BMS, 0.3 μM), NVP-AEW541 (NVP, 6 μM), LY294002 (LY, 5 μM) and wortmannin (WORT, 0.06 μM). (**c**) The experiment was performed as described in (**a**). Inhibitors used are Akti-1/2 (Akti, 5 μM), or MK2206 (MK, 2 μM). Values shown in **(b)** and **(c)** are mean ± SEM. n = 10–26. Different letters indicate statistically significant differences at *p* < 0.05.

We have previously shown that low [Ca^2+^] stress activates PI3K-Akt signaling in NaR cells exclusively in an IGF1R-dependent manner^25^. In zebrafish, there are two *igf1r* genes and two *insr* genes due to a teleost linage-specific genome duplication and these genes are expressed ubiquitously in embryonic and larval tissues^29, 30^. To definitely show that these *igf1r* and *insr* genes are expressed in NaR cells and to determine whether their mRNA levels change in response to low [Ca^2+^] stress, *Tg*(*igfbp5a*:*GFP*) larvae were transferred to normal and low [Ca^2+^] solution, NaR cells were isolated by FACS-based cell sorting. qPCR analysis showed that *igf1ra*, *igf1rb*, *insra*, and *insrb* mRNA were all detected and their levels did not change under low [Ca^2+^] stress (Supplemental Fig. S6). In a previous study, we have shown that Igfbp5a knockdown abolishes low [Ca^2+^]-induced Akt signaling in NaR cells^25^, suggesting the involvement of IGF1R^25^. We used a selective IGF1R inhibitor BMS-754807 to further investigate the role of IGF1R signaling. BMS-754807 treatment abolished low [Ca^2+^]-induced NaR cell proliferation in *Tg*(*igfbp5a*:*GFP*) larvae (Fig. 6a). NVP-AEW541, a structurally different IGF1R inhibitor, had a similar effect (Fig. 6b). The addition of LY294002 and wortmannin, two distinct PI3K inhibitors, abolished low [Ca^2+^]-induced NaR cell proliferation (Fig. 6b). Likewise, the addition of Akt inhibitors Akti-1/2 or MK2206 significantly inhibited low [Ca^2+^] stress-induced NaR cell proliferation (Fig. 6c). Collectively, these results further support the critical role of the IGF1R-PI3K-Akt signaling in low [Ca^2+^]-induced NaR cell proliferation.

**Figure 7:**
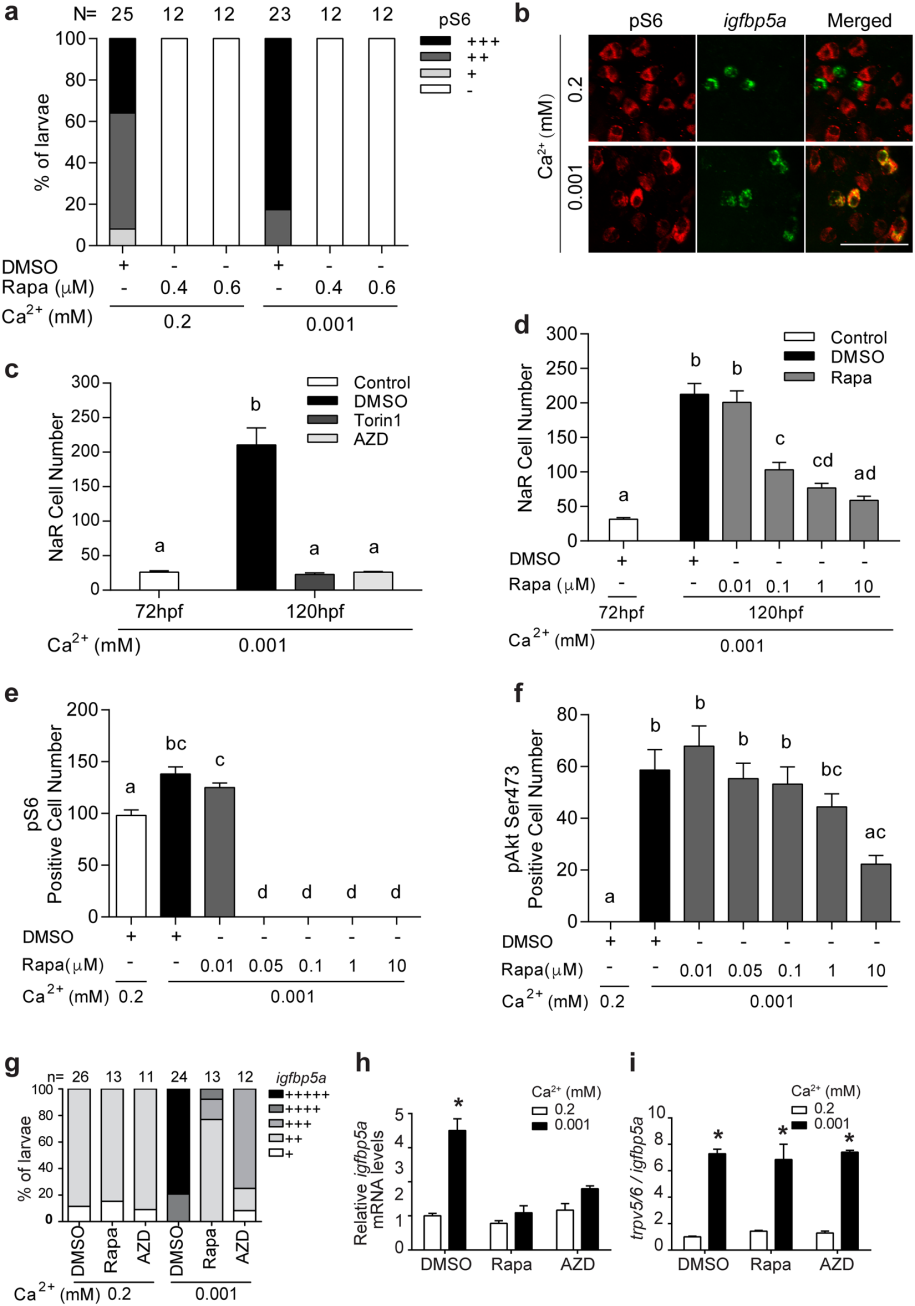
Activation of Tor signaling in NaR cells is required for their proliferation under low [Ca^2+^] stress. (**a**) Low [Ca^2+^] stress increases Tor signaling. 72 hpf wild type larvae were transferred to the indicated solutions. After 24 hours, they were stained using a pS6 antibody, and the results were quantified following the score system shown in Supplemental Fig. S7a. The total numbers of larvae are shown on the top of each column. (**b**) Low [Ca^2+^] activates Tor signaling in NaR cells. Larvae described in (**a**) were analyzed by pS6 immunostaining (red, left panel) and *in situ* hybridization for *igfbp5a* mRNA expression (green, middle panel). Merged views are shown in the right panel. (**c**) Tor inhibition abolishes NaR cell proliferation under low [Ca^2+^] stress. 72 hpf *Tg*(*igfbp5a*:*GFP*) larvae were treated and analyzed as described in Fig. 6. Inhibitors used are Torin1 (1 μM) and AZD8055 (AZD, 1.25 μM). (**d**) Dose-dependent effects of rapamycin (Rapa). Data shown are mean ± SEM, n = 10–13. Different letters indicate statistically significant differences at *p* < 0.05. (**e**,**f**) Different effects of rapamycin on Tor and pAkt signaling. Wild type larvae (72 hpf) were transferred to 0.2 mM or 0.001 mM [Ca^2+^] embryo rearing solution containing the indicated doses of rapamycin. 24 hours later, they were stained using anti pS6 (**e**) or pAkt antibodies (**f**). Values shown in **(b**–**f)** are mean ± SEM. Different letters indicate significant differences at *p* < 0.05, n = 10–15. (**g**–**i)** Inhibition of Tor signaling abolishes NaR cell proliferation (**g**) and *igfbp5a* mRNA expression (**h**) but not *trpv5*/*6* mRNA expression (**i**). Wild type larvae (72 hpf) were transferred to 0.2 mM or 0.001 mM [Ca^2+^] embryo rearing solution containing DMSO, rapamycin (0.6 μM) or AZD8055 (1 μM), and raised to 120 hpf. NaR cells were labeled by *in situ* hybridization for *igfbp5a* mRNA and scored according to a published scoring system^25^. The total numbers of larvae analyzed are shown on the top. The mRNA levels of *igfbp5a* (**h**) and *trpv5*/*6* (**i**) were measured by qPCR and normalized by the *β-actin* and *igfbp5a* mRNA levels, respectively. Values shown are mean ± SEM, n = 3. *p < 0.001 compared with the corresponding normal [Ca^2+^] control group.

Next, the possible involvement of Tor signaling was investigated. A considerable number of cells in the yolk sac region were positive for pS6 staining in the normal [Ca^2+^] group, indicating a relatively high basal level of Tor signaling (Fig. 7a). The pS6 signal was specific because it was abolished by rapamycin (Fig. 7a). The number of cells with strong pS6 signal was greater in the low [Ca^2+^] group (Fig. 7a), suggesting elevated Tor signaling under low [Ca^2+^] stress. Further analysis revealed that 98% (41 out of 42 cells) of pS6-positive cells in the low [Ca^2+^] group were NaR cells, while none of the pS6-positive cells (0/44) in the normal [Ca^2+^] group were NaR cells (Fig. 7b). These data suggest that low [Ca^2+^] stress activates Tor signaling in NaR cells specifically.

Tor inhibitors AZD8055 and Torin1 competitively target the ATP binding site to impede kinase activity of both Torc1 and Torc2^31^. Torin1 and AZD8055 treatment abolished low [Ca^2+^]-induced NaR cell proliferation (Fig. 7c). Treatment of larvae with Torin1 and AZD8055 also blocked the low [Ca^2+^]-induced increase in pAkt-positive cell number (Supplemental Fig. S7). To clarify the roles of Torc1 and Torc2, *Tg*(*igfbp5a*:*GFP*) larvae were treated with different concentrations of rapamycin, which preferentially inhibits Torc1^32^. Rapamycin inhibited low [Ca^2+^]-induced NaR cell proliferation at concentrations as low as 0.1 μM (Fig. 7d). Immunostaining showed that rapamycin completely abolished pS6 signal at 0.05 μM and higher (Fig. 7e). However, it did not affect pAkt levels at concentrations as high as 1 μM (Fig. 7f). Similar results were obtained in wild type larvae (Fig. 7g). Inhibition of Tor signaling by rapamycin or AZD8055 abolished the increased *igfbp5a* mRNA expression (Fig. 7h), whereas it had no effect on the elevated *trpv5*/*6* expression under low [Ca^2+^] stress (Fig. 7i). These results suggest that both Torc1 and Torc2 are involved; while Torc2 acts by phosphorylating Akt, Torc1 plays a more direct role in NaR cell proliferation under low [Ca^2+^] stress.

## Discussion

In this study, we have generated a stable transgenic zebrafish line expressing GFP in NaR cells under the control of the *igfbp5a* promoter. *Tg*(*igfbp5a*:*GFP*) larvae faithfully recapitulate NaR cell development and low [Ca^2+^]-induced NaR cell proliferation. Real-time imaging revealed that newborn NaR cells are mainly derived from pre-existing NaR cells. NaR cells undergo at least two cell divisions during the 48 h treatment period. While the first cell division is completed around 24 h after the treatment, the second is completed 12 h later. These data suggest that low [Ca^2+^] stress promotes NaR cell proliferation by re-activating a mitotic program in pre-exiting NaR cells. Importantly, the low [Ca^2+^]-induced NaR cell proliferation is dependent on IGF1R-PI3K-Akt-Tor signaling activation. The *Tg*(*igfbp5a*:*GFP*) larvae can be readily adapted for automated high-throughput analysis because changes in NaR cell number can be determined by measuring GFP fluorescence.

In the zebrafish genome, there are two *igf1r* genes and two *insr* genes due to a teleost linage-specific genome duplication^29, 30^. The results of this study showed that all 4 genes are expressed in NaR cells but their levels did not change under low [Ca^2+^] stress. The low [Ca^2+^]-induced pAkt signaling and NaR cell proliferation are likely mediated through the IGF1Rs. This notion is supported by the following facts: (1) two structurally distinct and selective IGF1R inhibitors blocked low [Ca^2+^]-induced pAkt signaling and NaR cell proliferation; (2) knocking down of Igfbp5a abolishes low [Ca^2+^]-induced Akt signaling in NaR cells^25^. Studies in mammalian systems have shown that IGFBP5 has several modes of action. IGFBP5 can modulate local IGF activities by inhibiting or enhancing the IGF activities or can even have ligand-independent action^33–35^. In differentiating mouse myoblasts, for example, IGFBP5 is located on the cell surface^35^. The cell surface-associated IGFBP5 binds to IGF-2 and brings IGF-2 into close proximity of the IGF1R, thereby enhancing IGF1R-mediated PI3K-Akt signaling activity^35^. In NaR cells, locally expressed Igfbp5a may act in a similar fashion to facilitate the IGF ligand–receptor interaction. Future studies will be needed to elucidate the specific mechanism(s) by which Igfbp5a promote IGF signaling in NaR cells.

In this study, we have shown that several structurally distinct inhibitors targeting PI3K and Akt all blocked low [Ca^2+^]-induced NaR cell proliferation. These effects are unlikely due to any potential indirect effects on calcium signaling because we have shown that BAPTA-AM, an intracellular Ca^2+^ chelator, did not affect the low [Ca^2+^]-induced NaR cell proliferation^25^. Likewise, addition of the L-type calcium channel blocker verapamil and two calmodulin antagonists (W7 and calmidazolium) had no effect^25^. However, we cannot exclude the possibility that one or more of these IGF1R, PI3K, and Akt inhibitors may indirectly alter intracellular calcium levels in NaR cells. Future *in vivo* calcium imaging studies will be needed to clarify this issue.

A new finding made in this study is that low [Ca^2+^] stress activates Tor signaling in NaR cells specifically and that Tor signaling is required for low [Ca^2+^]-induced NaR cell proliferation. Although there were considerable levels of pS6-positive cells on the yolk sac under normal [Ca^2+^] condition, these cells were not NaR cells since they do not express *igfbp5a* mRNA. Low [Ca^2+^] stress caused a marked increase in pS6-positive cells in the yolk sac region, and 98% of these pS6-positive cells in the low [Ca^2+^] group were NaR cells. This conclusion is also in good agreement with our previous study showing that low [Ca^2+^] stress activates causes PI3K-Akt signaling in NaR cells exclusively^25^. Addition of Torin1, AZD8055, and rapamycin all abolished NaR cell proliferation under low [Ca^2+^]. TOR is the catalytic subunit of TORC1 and TORC2^10^. TORC1, consisting of TOR, Raptor, MLST8, PRAS40, and DEPTOR, regulates protein synthesis^36^. TORC2 is composed of TOR, RICTOR, mSIN1, DEPTOR, TTI1 and TEL2. It phosphorylates Akt/PKB at S473, which in turn leads to Akt phosphorylation by PDK1 at T308 and results in full Akt activation. The activated Akt then activates TORC1^36^. Using *Tg*(*igfbp5a*:*GFP*) larvae, we showed that Torc2 contributes to NaR cell proliferation by phosphorylating Akt, while Torc1 plays a more direct role in NaR cell proliferation under low [Ca^2+^] stress. This conclusion is supported by the following lines of evidence: (1) treatment of zebrafish larvae with Torin1 or AZD8055 blocked low [Ca^2+^] stress-induced increase in pAkt at S473. Torin1 and AZD8055 treatment also abolished low [Ca^2+^] stress-induced NaR cell proliferation; (2) rapamycin inhibited low [Ca^2+^] stress-induced NaR cells at concentrations as low as 0.1 μM. At this concentration, rapamycin completely abolished the pS6 signal, while it did not affect pAkt signaling.

When subjected to low [Ca^2+^] environments, many fishes, including zebrafish, show elevated Ca^2+^ transport capacity^37^. This enhanced Ca^2+^ uptake capability is critical for maintaining Ca^2+^ homeostasis and is achieved by elevated *trpv5*/*6* expression and increased NaR cell number^37^. The signaling mechanisms underlying these two adaptive responses are poorly understood. Our results suggest that the IIS-PI3K-Akt-Tor signaling plays a critical role in the increase in NaR cell number. The mitogenic action of IGF1R-PI3K-Akt-Tor signaling in NaR cells is likely due to its ability in regulating cell cycle regulators. It has been shown in mammalian cells that the PI3K-Akt-mTOR pathway elevates cyclin D1 level by increasing its translation through mTOR^38^ and by enhancing its stability and translocation into the nucleus through inhibition of GSK3B^39, 40^. Akt can also down-regulate CDKN1B through FOXO1^41^. Activation of the IGF1R-PI3K-Akt-Tor pathway, however, does not play a major role in the low [Ca^2+^]-induced up-regulation of *trpv5*/*6* expression in NaR cells. This conclusion is based on the following lines of evidence: (1) low [Ca^2+^] stress resulted in a similar degree of increases in *igfbp5a* mRNA levels and NaR cell number, but the increase in *trpv5*/*6* mRNA levels was more than 10-fold higher; (2) although low [Ca^2+^] treatment induces *trpv5*/*6* expression in both embryonic and larval stages, it only stimulates NaR cell proliferation and *igfbp5a* expression in the larva stage; (3) when switching from the normal [Ca^2+^] to the low [Ca^2+^] solution, the levels of *trpv5*/*6* increased much quicker compared to those in *igfbp5a* mRNA and NaR cell numbers; finally (4) inhibition of Tor signaling abolished the increased *igfbp5a* mRNA expression and NaR cell proliferation, while it had no effect on the elevated *trpv5*/*6* expression under low [Ca^2+^] stress. These findings indicate that the elevated *trpv5*/*6* mRNA levels are due to the increased NaR cell number as well as an increased *trpv5*/*6* expression per cell, and the latter change occurs faster. The mechanism(s) underlying low [Ca^2+^] stress-induced rapid increase in *trpv5*/*6* gene expression in NaR cells are yet to be elucidated. 1,25-(OH)_2_D_3_ has been shown to increase *TRPV5* and/or *TRPV6* expression at the transcriptional levels in mammals^42, 43^, likely through the vitamin D receptor response elements (VDREs) on the gene promoter^44, 45^. In zebrafish, knockdown of VDRa decreased the *trpv5*/*6* mRNA expression^46^. It will be of interest to determine the possible role of vitamin D in regulating *trpv5*/*6* expression in zebrafish NaR cells.

In summary, we have developed a whole organism and phenotype-based platform that faithfully reports the mitotic action of IGF1R-PI3K-Akt-Tor signaling in epithelial cells in real-time. This platform is well suited for high-throughput analysis and real-time cell cycle analysis. Using this platform, the dynamics of low [Ca^2+^]-induced epithelial cell proliferation was determined, and the *in vivo* roles of Torc1 and Torc2 in epithelial cell proliferation elucidated. Because of their tiny size, optical clarity, and since NaR cells are located on the yolk sac skin and readily take up chemical compounds, these *Tg*(*igfbp5a*:*GFP*) larvae can be readily used for chemical biology screens to identify novel players and inhibitors of the IGF1R-PI3K-Akt-Tor pathway.

## Materials and Methods

### Chemicals and reagents

All chemicals and reagents were purchased from Fisher Scientific (Pittsburgh, PA) unless otherwise specified. NVP-AEW541 was obtained from Novartis (Basel, Switzerland) and BMS-754807 was purchased from JiHe Pharmaceutica (Beijing, China). Wortmannin and LY294002 were purchased from Cell Signaling Technology (Danvers, MA). AZD8055 and Torin1 were purchased from Selleck Chemicals (Houston, TX). Rapamycin and Akti-1/2 were purchased from Calbiochem (Gibbstown, NJ). MK2206 was purchased from ChemieTek (Indianapolis, IN). All inhibitors were dissolved in DMSO and further diluted in water. Restriction enzymes were purchased from New England Bio Labs (Beverly, MA). The TRIzol reagent, M-MLV reverse transcriptase, and RNaseOUT™ Recombinant Ribonuclease Inhibitor were purchased from Invitrogen (Waltham, MA). The anti-GFP antibody was purchased from Torrey Pines Biolabs (Secaucus, NJ). The Phospho-Akt (Ser 473) and Phospho-S6 antibody were purchased from Cell Signaling Technology (Beverly, MA).

### Experimental animals

Zebrafish were maintained at 28 °C under a light/dark cycle of 14/10 hours and fed twice daily. Fertilized eggs were obtained by natural breeding. They were raised and staged as described previously^47^. Embryos were raised in embryo rearing solution supplemented with 0.003% (w/v) 2-phenylthiourea N-phenylthiourea (PTU). Embryo rearing solution containing various concentrations of [Ca^2+^] was prepared as previously reported^25^. All experiments were conducted in accordance with the guidelines approved by the Institutional Committee on the Use and Care of Animals, University of Michigan at Ann Arbor.

### BAC DNA construction

The *BAC*(*igfbp5a*:*GFP*) construct was generated following Suster *et al*.^48^. Briefly, a BAC clone (DKEYP-56B7), which contains zebrafish *igfbp5a* genome DNA, was selected from the DanioKeypilot library (Source BioScienc, Nottingham, UK). DKEYP-56B7 DNA was transformed into SW102 cells and verified by restriction enzyme digestion. Next, the *iTol2* cassette and *GFP-Kanamycin resistance* gene cassette were recombined into the BAC. The primers used to amplify the GFP cassette sequence are: igfbp5a_GFP_fw, 5′-GTTTTGCCATTTCAAAGCTGGTGAAATAGGTGTTCTACAGTAGGACGATGGTGAGCAAGGGCGAGGAGCTGTTC-3′ and igfbp5a_frt-kan-rev, 5′-GTTTACTTTTGTCCCATATAAAACAAATACTACAAGTCAATAAAACATACCCGCGTGTAGGCTGGAGCTGCTTC-3′. The resulted *BAC*(*igfbp5a*:*GFP*) was validated by PCR using the following primers: igfbp5a-5UTR-fw, 5′-GAACGCTGTTCGCTTGAT-3′ and GFP-rev, 5′-GCTGAACTTGTGGCCGTTTACG-3′.

### Microinjection and generation of the transgenic fish line

The *BAC*(*igfbp5a*:*GFP*) DNA and *Tol2* mRNA were mixed and injected into 1-cell stage zebrafish embryos. The injected embryos were placed in normal [Ca^2+^] embryo rearing solution and kept at 28.5 °C. The F0 embryos were screened at 72 hpf by checking GFP fluorescence. GFP-positive F0 embryos were raised and crossed with wild type fish to obtain F1 individuals. The F1 heterozygous fish were identified by GFP fluorescence, and genotyped by ADL-PCR. The F1 heterozygous adult fish carrying same integration site were in-crossed to obtain F2 individuals. F2 homozygous fish were identified by crossing with wild type fish and checking the ratio of GFP-positive offspring.

### Genotyping of *Tg*(*igfbp5a*:*GFP*)

The ADL-PCR was performed following a published protocol^48^. Briefly, the adaptor was made by annealing ASS primer (5′-GATCACCTGCCCCCGCTT-3′) and ALS primer (5′-CTAATACGACTCACTATAGGGCTCGAGCGGCCGCGGGGGCAGGT-3′). The genomic DNA was extracted and digested by Sal 1 at 37 °C for 0.5 h. The adaptor was ligated to the digested genomic DNA. The ligation product was used as a template in the first round PCR using primer Ap1 (5′-GGATCCTAATACGACTCACTATAGGG-3′) and primer 150R-out (5′-AATACTCAAGTACAATTTTA-3′). The PCR product (1:10 dilution) was used as a template for the second round of PCR. Primer Ap2 (5′-CACTATAGGGCTCGAGCGG-3′) and 100R-out (5′-AGATTCTAGCCAGATACT-3′) were used in the second round of PCR. The final PCR product was sequenced at the University of Michigan DNA Sequencing Core Facility.

### Whole-mount *in situ* hybridization and immunostaining

Single color *in situ* hybridization was performed as previously reported^47^. Double-color *in situ* hybridization and immunofluorescence co-staining were conducted as previously described^25^. Briefly, *igfbp5a* mRNA and *trpv5*/*6* mRNA were detected using a digoxigenin (DIG)-labeled antisense riboprobe. After incubation in an anti-DIG-POD antibody (Perkin Elmer. MA), larvae were stained in Alexa 488-tyramide substrate. After staining, they were washed in 1xPBST and incubated with an anti-GFP antibody overnight at 4 °C. Next, they were stained with a Cy3 conjugated Goat anti-Rabbit IgG antibody. Fluorescent images were acquired using a Leica TCS SP5 confocal microscope with the Leica LAS AF software.

### Reverse transcription (RT) and quantitative real-time PCR (qPCR)

Total RNA was isolated from embryos using TRIzol reagent and reverse-transcribed to cDNA using M-MLV reverse transcriptase (Invitrogen, Carlsbad, CA). qPCR was carried out using iQ SYBR Green Supermix (Bio-Rad) with an iCycler iQ Multicolor real-time PCR detection system (Bio-Rad, Hercules, CA). The efficiency and specificity of the qPCR were verified by the standard curve and denaturing curve analyses. The expression level of a particular gene transcript was calculated based on the standard curve and normalized to *β-actin* or 18S RNA level. The following primers were used (*igfbp5a*: 5′-GCTGCACGCTCTGCTTTAC-3′ and 5′-AATGGAACCTTGGCCTGAG-3′; *trpv5*/*6*: 5′-GGACCCTACGTCATTGTGATAC-3′ and 5′-GGTACTGCGGAAGTGCTAAG-3′; *β-actin*: 5′-GATCTGGCATCACACCTTCTAC-3′ and 5′-CCTGGATGGCCACATACAT-3′; *igf1ra*: 5′-CGTACCTCAATGCCAACAAG-3′ and 5′-TAGGGCTGTTCGGCTAATGT-3′; *igf1rb*: 5′-AAACTTGGGACCAGGGAACT-3′ and 5′-ATCTTCTCCCGCTCCACTTC-3′; *insra*: 5′-CAACATGCCCCCTCACCACT-3′ and 5′-CGACACACATGTTGTTGTG-3′; *insrb*: 5′-GACTGATTACTATCGCAAGGG-3′ and 5′-TCCAGGTATCCTCCGTCCAT-3′; *18s*: 5′-AATCGCATTTGCCATCACCG-3′ and 5′-TCACCACCCTCTCAACCTCA-3′).

### Flow Cytometry Analysis and Fluorescence-activated cell sorting (FACS)

For Flow cytometry analysis, *Tg*(*igfbp5a*:*GFP*) larvae were transferred to 0.2 or 0.001 mM [Ca^2+^] water for at 72 hpf and sampled at 120 hpf. Wild type larvae of the same stage were used as negative controls. 40 larvae in each group were digested with Liberase TM (0.28 Wünsch units/ml in HBSS) for 1 h at 28.5 °C. The digestion reaction was stopped by adding fetal calf serum (5% v/v). The dissociated cells were collected by centrifugation, washed three times in HBSS, and stained with Vybrant® DyeCycle™ Violet (1:1000) for 1 h. They were then subjected to Flow Cytometry Analysis in an Attune Acoustic Focusing Cytometer (Applied Biosystems, Life Technologies, Carlsbad, CA). A two-parameter dot-plot of VL1 (DyeCycle™ Violet) area (VL1-A) versus VL1 width (VL1-W) was constructed to exclude unstained cells and cell clumps. A two-parameter dot-plot of FSC-A versus BL1 height (GFP) (BL1-H) was constructed to gate GFP-positive cells. A single-parameter VL1-area histogram was constructed to illustrate relative DNA content.

For FACS sorting, 200 *Tg*(*igfbp5a*:*GFP*) larvae (72 hpf) were transferred to 0.2 mM or 0.001 mM [Ca^2+^] water for 18 h. Single cell suspension was made as described above. NaR cells were sorted using FACSAria cell sorter (BD Biosciences, Franklin Lakes, NJ). Wild type larvae with the same treatment were used as negative control to set the sorting gate of GFP fluorescent signal. The gate was set so that less than 0.2% of cells from wild type were GFP positive cells (GFP+). The sorted cells (~10,000 cells) were directly collected into 75 μL cell lysis buffer provided with RNeasy Micro kit (Qiagen, Valencia, CA) followed by RNA isolation. 22 ng total RNA was reverse transcribed using SuperScript III Reverse Transcriptase. RNaseOUT™ Recombinant Ribonuclease Inhibitor was added into the reverse transcript system to protect RNA from degradation. qPCR was carried out as described above.

### Live imaging of *Tg*(*igfbp5a*:*GFP*) transgenic fish

*Tg*(*igfbp5a*:*GFP*) larvae (72 hpf) were immobilized using low melting point (LMP) agarose gel. Mounting solution was prepared by dissolving 0.5% (w/v) LMP agarose in normal [Ca^2+^] or low [Ca^2+^] solutions containing Tricaine and PTU. The immobilized larvae were placed in a petri dish and submerged with normal [Ca^2+^] or low [Ca^2+^] embryo rearing solution containing Tricaine and PTU. Lateral views were taken hourly from 72 to 120 hpf using a Leica MZ16F stereo microscope (Leica, Hamburg, Germany) equipped with a QICAM 12-bit Mono Fast 1394 Cooled camera (QImaging, Surry, BC, Canada). QCapture Pro Version 6.0.0 software was used. The time-lapse video was generated using Windows Movie Maker (http://www.windows-movie-maker.org/).

### Statistical analysis

Values are presented as mean ± standard error of the mean (SEM). Statistical significance among experimental groups was determined using one-way ANOVA followed by Tukey’s multiple comparison test. Statistical tests were performed using GraphPad Prism 6 software (GraphPad Software, Inc., San Diego, CA), and significance was defined as *p* < 0.05 or greater. Correlation analysis was carried out by fitting the data to the equation Y = X to calculate *r* ^*2*^ using GraphPad Prism 6.

## Electronic supplementary material

Supplementary information

Supplementary Video
