# Supplementary material for: Development of a Whole Organism Platform for Phenotype-Based Analysis of IGF1R-PI3K-Akt-Tor Action
Source: Sci Rep. 2017 May 17;7:1994. doi: 10.1038/s41598-017-01687-3 (PMC5435685; doi:10.1038/s41598-017-01687-3)

Development of a Whole Organism Platform for Phenotype-Based Analysis of IGF1R-PI3K-Akt-Tor Action

Chengdong Liu[1,2], Wei Dai[2#], Yan Bai[2], Changfeng Chi[2,3], Yi Xin[2], Gen He[1], Kangsen Mai[1], and Cunming Duan[2*]

1) The Key Laboratory of Mariculture, Education Ministry of China and College of Fisheries, Ocean University of China, Qingdao, 266003, China;

2) Department of Molecular, Cellular and Developmental Biology, University of Michigan, Ann Arbor, MI 48109; and

3) National Engineering Research Center for Marine Facilities Aquaculture, School of Marine Science and Technology, Zhejiang Ocean University, Zhoushan, 316022, China

Wei Dai, Present address: Department of Molecular, Cellular and Developmental Biology, University of California, Santa Barbara, CA 93106

**** Corresponding author and person to whom reprint requests should be addressed:***

Cunming Duan

Department of Molecular, Cellular, and Developmental Biology

University of Michigan

Ann Arbor, MI 48109

cduan@umich.edu

Supplemental figure legends

Supplemental Figure 1. Low [Ca^{2+}] stress increases the *trpv5/6* expression but not NaR cell number or *igfbp5a* mRNA expression in early embryos. **a-b**) Zebrafish embryos were raised in normal or low [Ca^{2+}] embryo rearing solution from 0 to 48 and 72 hpf. The levels of *trpv5/6* (**a**) and *igfbp5a* (**b**) mRNA were measured and normalized by *β-actin* mRNA levels. Data shown are mean ± SEM, n = 3. Different letters indicate differences at $p < 0.05$. **c**) 72 hpf embryos raised in the indicated [Ca^{2+}] water were analyzed by *in situ* hybridization using *igfbp5a* probe. NaR cells on both side of yolk sac were manually counted. Data shown are mean ± SEM, n = 8-9. ns, not statistically significant.

Supplemental Figure 2. Different effects of low [Ca^{2+}] stress on *igfbp5a* and *trpv5/6* mRNA levels. **a**) Schematic diagram illustrating the experimental design. **b-c**) Changes in *trpv5/6* (**b**) and *igfbp5a* (**c**) mRNA levels. Data show are Mean ± SEM, n=3. Different letters indicate statistically significant differences at $p < 0.05$.

Supplemental Figure 3. Generation of *Tg(igfbp5a:GFP)* transgenic fish using Tol2-mediated BAC transgenesis. **a**) Schematic diagram of the *BAC(igfbp5a:GFP)* construct engineered. Filled boxes indicate *igfbp5a* ORF and open boxes indicate UTR. The iTol2 cassette and GFP reporter gene cassette were introduced into DKEYP-56B7 by homologous recombination. The *igfbp5a* sequence from the start codon to the end of first exon was replaced by the GFP cassette. **b**) The indicated DNA was subjected to Sal I digestion. Arrows showed the different bands after recombineering. **c**) PCR validation of the GFP cassette insertion. Forward and reverse primers were designed to target the 5'UTR of *igfbp5a* and the *GFP* gene protein coding sequence (upper panel). PCR result is shown in the lower panel. **d)** Representative view of GFP expression in *BAC(igfbp5a:GFP)*-injected F0 larvae at 120 hpf.

Supplemental Figure 4. The GFP-positive cells are NaR cells. **a)** GFP-positive cells express *trpv5/6* mRNA. *Tg(igfbp5a:GFP)* raised in normal $[Ca^{2+}]$ medium were sampled at 120 hpf and analyzed by *in situ* hybridization for *trpv5/6* mRNA expression (green, left panels) and GFP immunostaining (red, middle panel). Merged view is shown in the right panel. Scale bar = 50 µm. **b-c)** The *igfbp5a* mRNA (**b**) and *trpv5/6* mRNA (**c**) is highly expressed in GFP-positive cells . *Tg(igfbp5a:GFP)* larvae (72 hpf) were transferred to 0.2 or 0.001 mM $[Ca^{2+}]$ medium. 18h later, GFP-positive cells were isolated by FACS as described in Materials and Methods. The levels of *igfbp5a* and *trpv5/6* mRNA were determined by qPCR and normalized by the *18s* mRNA levels. Values are mean ± SEM of two independent experiments. GFP+ and GFP- indicates GFP positive- and GFP-negative cells.

Supplemental Figure 5. The GFP fluorescence intensity is highly correlated with NaR cell number. The NaR cell number and GFP fluorescence intensity data shown in Fig. 4 were subjected to correlation analysis.

Supplemental Figure 6. The expressions of 4 *igf1r* and *insr* mRNA in NaR cells. *Tg(igfbp5a:GFP)* larvae (72 hpf) were transferred to 0.2 or 0.001 mM $[Ca^{2+}]$ water for 18h. NaR cells were isolated using FACS. The expressions of *igf1ra* (**a**), *igf1rb* (**b**), *insra* (**c**) and *insrb* (**d**) mRNA were measured by qPCR and normalized by *18s* mRNA levels. Values are mean ± SEM. Values are mean ± SEM of two independent experiments.

Supplemental Figure 7. **a)** Four different levels of pS6 signal were observed in zebrafish larvae and used for quantification. **b)** Torin1 treatment abolishes low $[Ca^{2+}]$-induced pAkt signaling. 72 hpf wild type larvae were transferred to 0.2 or 0.001 mM $[Ca^{2+}]$ solution containing DMSO or Torin1 (1µM). After 8h, they were subjected to immunostaining using an anti-phospho-Akt

(S473) antibody. Data shown are mean ± SEM. n = 14-16. Different letters indicate significant differences at $p < 0.05$. **c)** AZD8055 treatment abolishes low [Ca^{2+}]-induced pAkt signaling. AZD8055 (1 μM) and rapamycin (10 μM) were used. Representative images are shown.

Supplemental video 1. A 72 hpf *Tg(igfbp5a:GFP)* larva was transferred to low [Ca^{2+}] water and time lapse movie was taken over the next 48 h. The frame focuses on the yolk sac region where the NaR cells are located.

Supplemental Figure 1

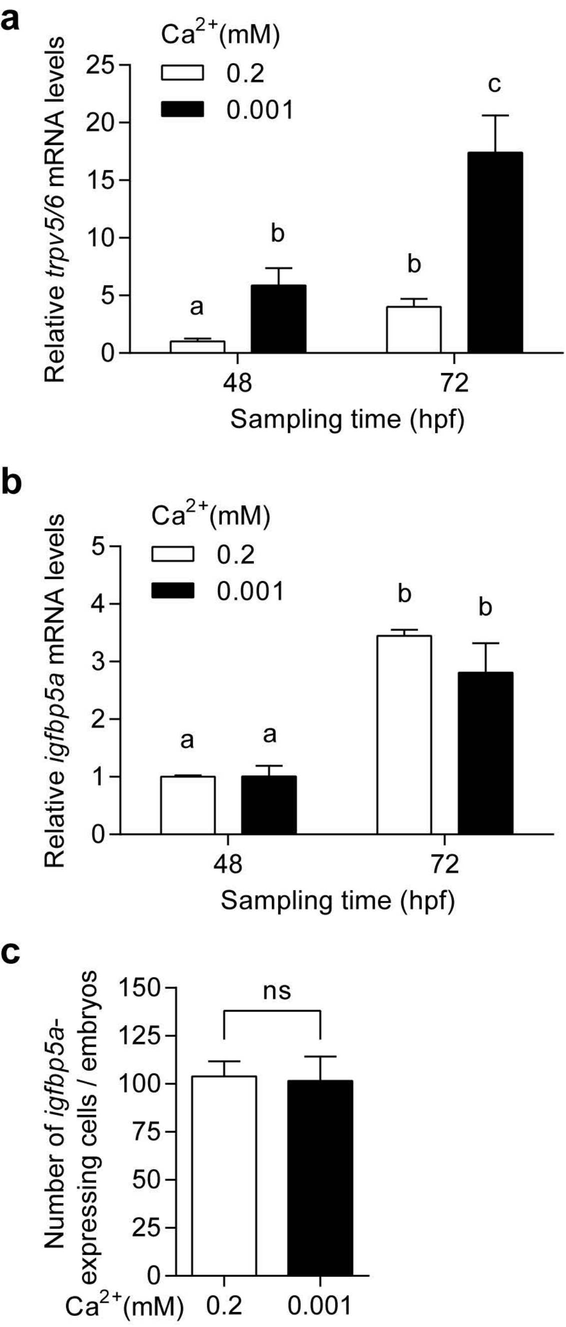

Supplemental Figure 2

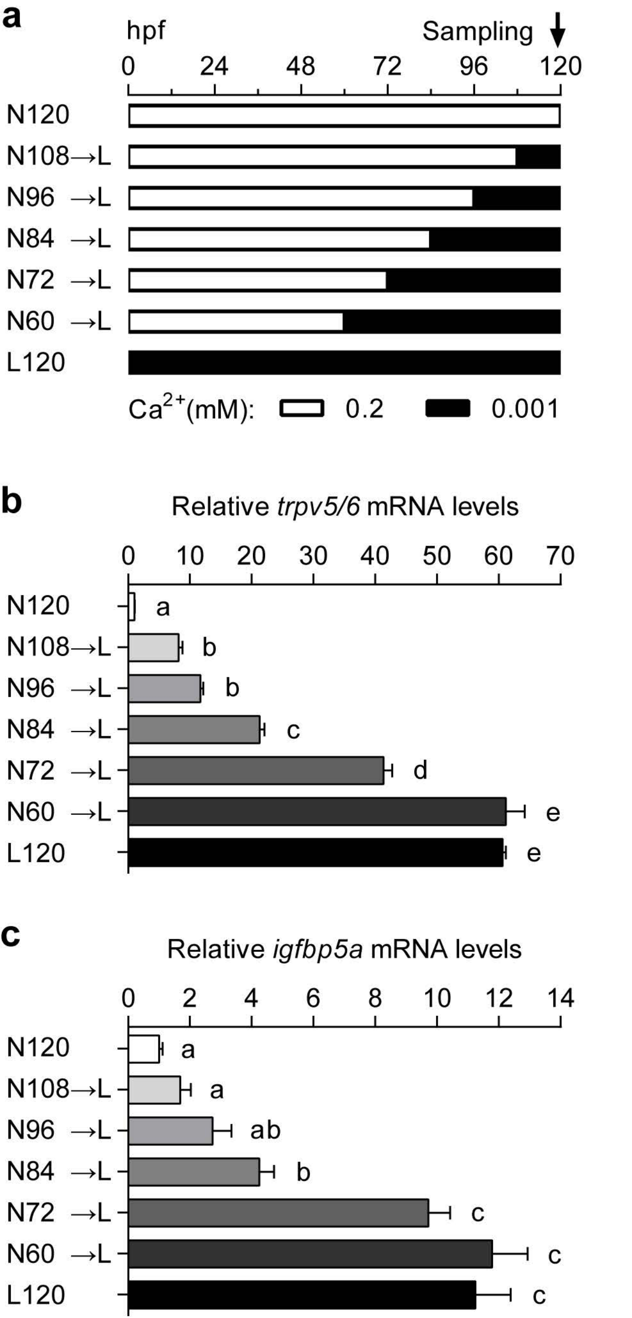

Supplemental Figure 3

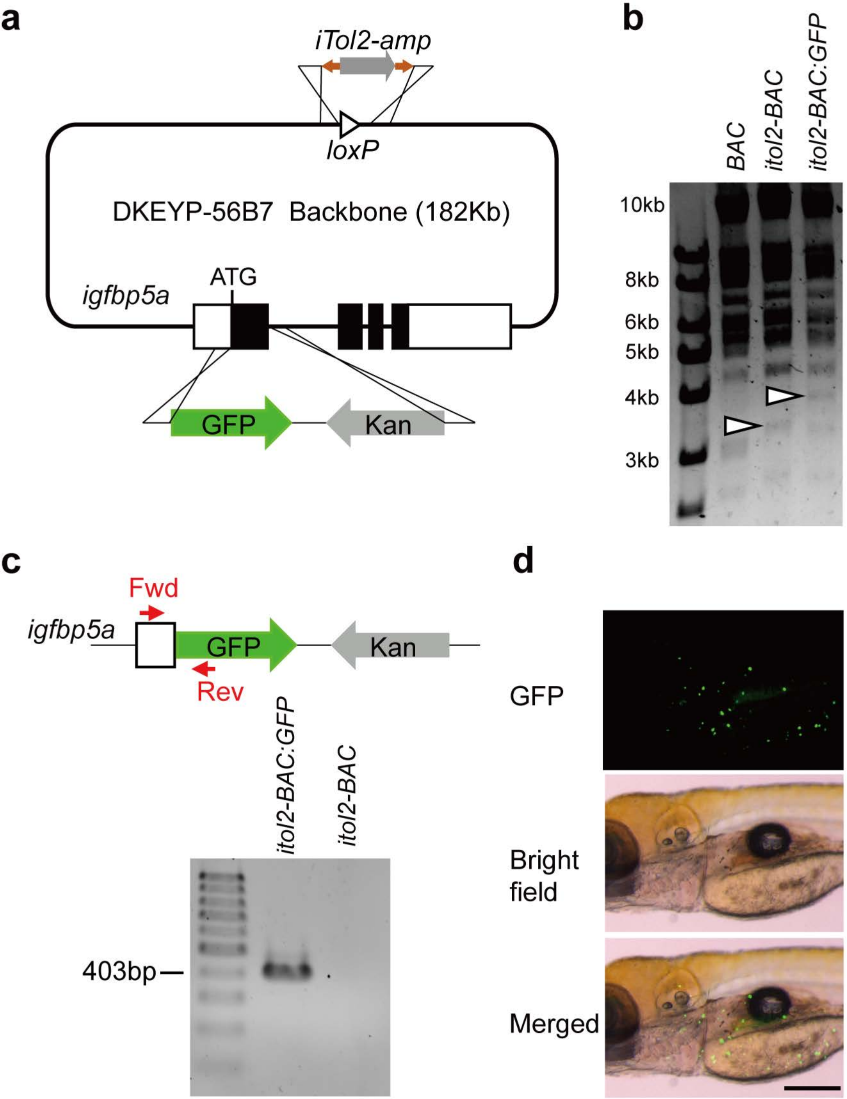

Supplemental Figure 4

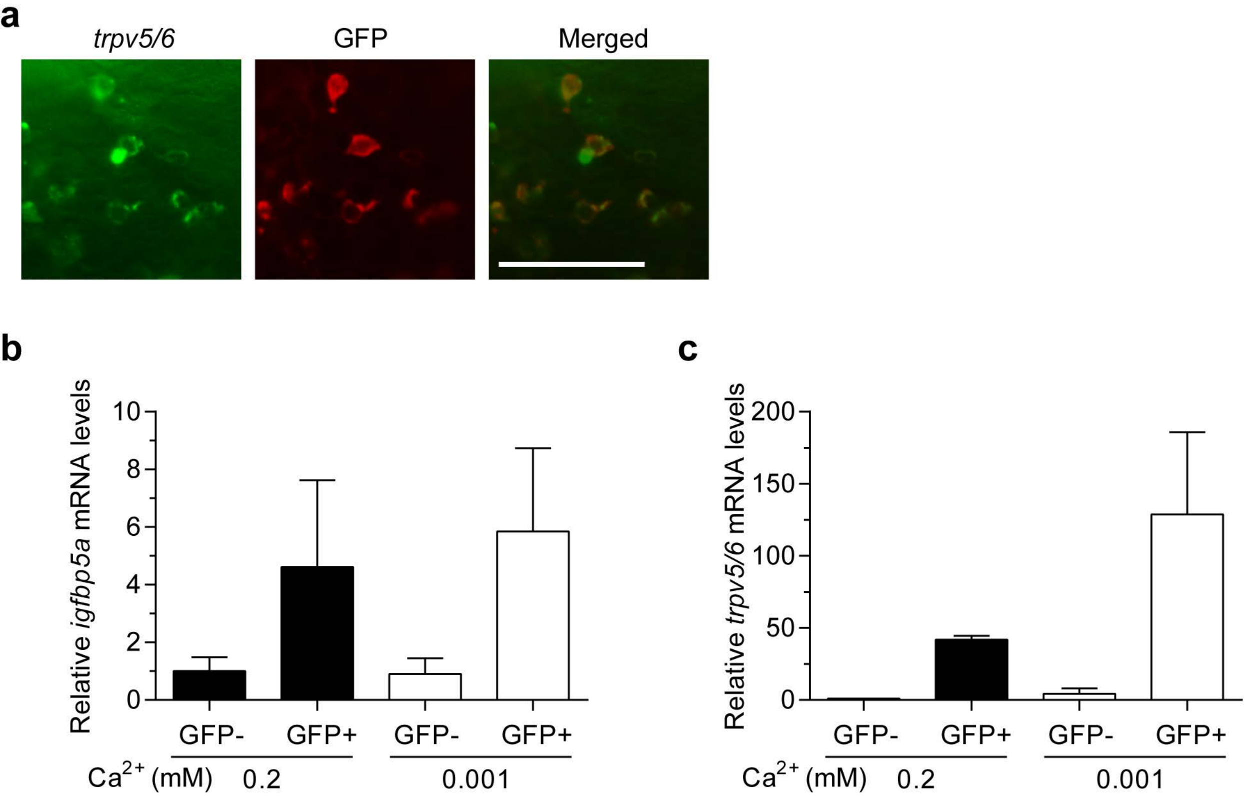

Supplemental Figure 5

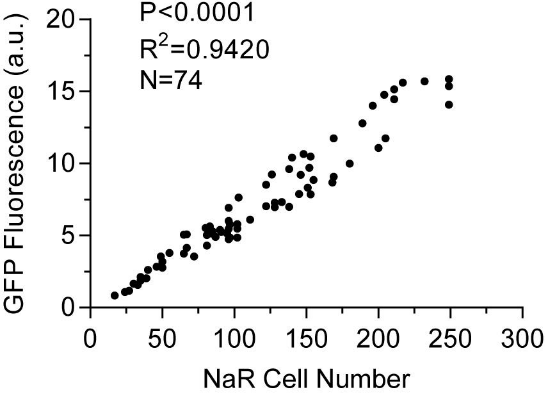

Supplemental Figure 6

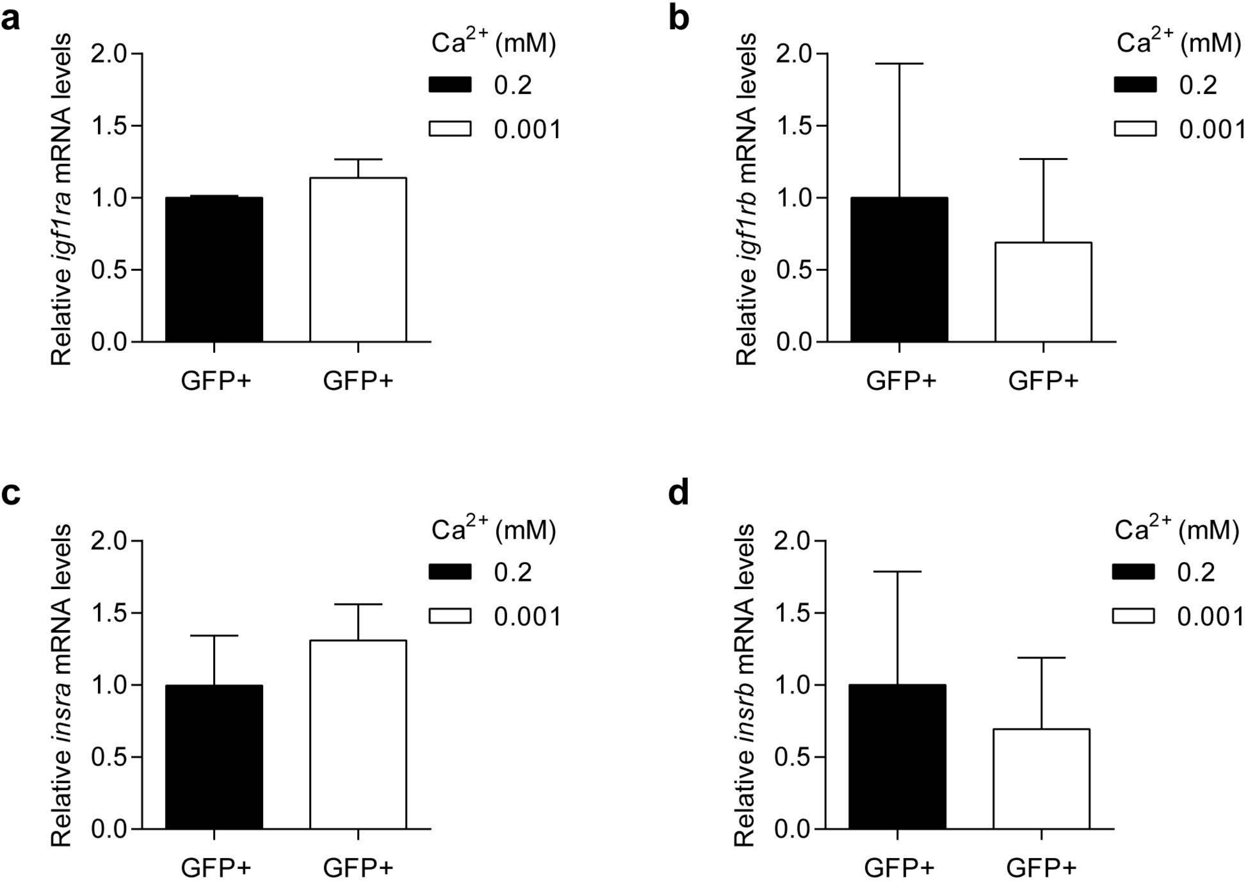

Supplemental Figure 7

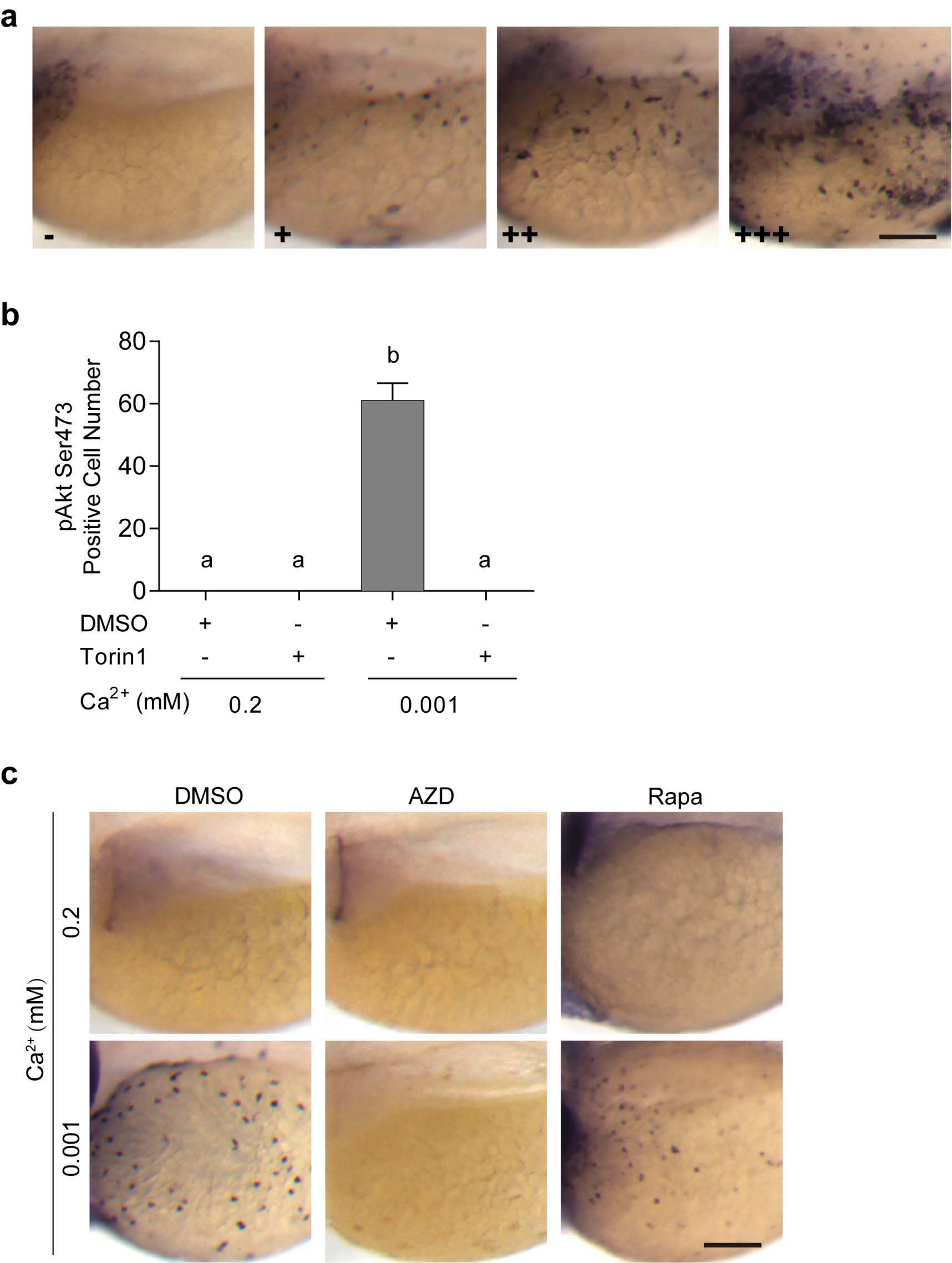

Supplement: Supplementary file 1 — Supplementary information [file 41598_2017_1687_MOESM1_ESM.pdf]
